# Mechanical Stress Impairs Mitosis Progression in Multi-Cellular Tumor Spheroids

**DOI:** 10.1371/journal.pone.0080447

**Published:** 2013-12-03

**Authors:** Annaïck Desmaison, Céline Frongia, Katia Grenier, Bernard Ducommun, Valérie Lobjois

**Affiliations:** 1 Université de Toulouse, ITAV-USR3505, Toulouse, France; 2 CNRS, ITAV-USR3505, Toulouse, France; 3 CNRS, LAAS-UPR81001, Toulouse, France; 4 CHU de Toulouse, Toulouse, France; University of California, San Diego, United States of America

## Abstract

Growing solid tumors are subjected to mechanical stress that influences their growth rate and development. However, little is known about its effects on tumor cell biology. To explore this issue, we investigated the impact of mechanical confinement on cell proliferation in MultiCellular Tumor Spheroids (MCTS), a 3D culture model that recapitulates the microenvironment, proliferative gradient, and cell-cell interactions of a tumor. Dedicated polydimethylsiloxane (PDMS) microdevices were designed to spatially restrict MCTS growth. In this confined environment, spheroids are likely to experience mechanical stress as indicated by their modified cell morphology and density and by their relaxation upon removal from the microdevice. We show that the proliferation gradient within mechanically confined spheroids is different in comparison to MCTS grown in suspension. Furthermore, we demonstrate that a population of cells within the body of mechanically confined MCTS is arrested at mitosis. Cell morphology analysis reveals that this mitotic arrest is not caused by impaired cell rounding, but rather that confinement negatively affects bipolar spindle assembly. All together these results suggest that mechanical stress induced by progressive confinement of growing spheroids could impair mitotic progression. This study paves the way to future research to better understand the tumor cell response to mechanical cues similar to those encountered during in vivo tumor development.

## Introduction

A tumor micro-region consists of a heterogeneous cancer cell population organized in a 3D structure in which cell growth is influenced by interactions with the microenvironment. The crosstalk between tumor cells and microenvironmental components, including the extracellular matrix (ECM), fibroblasts, endothelial and immune cells, is essential for tumor progression and drug resistance [1], [2]. In such complex environment, tumor growth and progression is influenced not only by biochemical parameters such as growth factors, cytokines, hormones or hypoxia, but also by mechanical cues [3], [4]. Indeed, sensing compression and tension forces (i.e., mechano-sensing) is an important component of cell physiology and changes in the mechanical homeostasis within tissues are observed during tumor growth [3], [5]. Cells sense forces through mechanoreceptors that are located at the plasma membrane and that transduce the information to the intracellular machinery to elicit a specific response to external mechanical cues [6]. Modification of the mechanical environment can modulate tumor cell growth [7], migration and invasion [7]–[10] as well as proliferation and apoptosis [11], [12].

One of the hallmarks of cancer cells is their ability to sustain uncontrolled proliferation through deregulation of cell cycle control mechanisms [2]. Many studies have contributed to deciphering the complex regulatory networks of proteins and biochemical signals that govern the progression of a cell through mitosis. Moreover, it has been demonstrated that mitosis progression is also mechanically regulated. Indeed, cell division is directed by the environment geometry and ECM organization [13], [14], requires cell rounding and depends on the interaction of the mitotic spindle with actin cytoskeleton components. However, the impact of mechanical cues on mitotic progression has been documented essentially using 2D monolayer-based models and very little is known about the consequence of mechanical stress on cell division within tumors.

Multicellular tumor spheroids (MCTS), in which cancer cells are cultured as 3D organized aggregates, are attractive models to investigate this issue. These complex multicellular systems reproduce the cell-cell and cell-matrix interactions found in solid tumors [15]. Moreover, MCTS can grow up to several hundred micrometers in diameter and progressively display a gradient of proliferating cells similar to what found in tumor micro-regions. Specifically, in large spheroids, dividing cells are in the outmost layers and quiescent cells are located more centrally in hypoxic and nutrient-poor regions [16], [17].

In this study, we used MCTS as experimental model to explore how a confined mechanical environment can affect tumor cell division within an organized tumor cell population. To this aim, we designed and produced dedicated polydimethylsiloxane (PDMS) microdevices that alter the microenvironment geometry and in which MCTS growth was mechanically confined. We show that such conditions do not impair cell rounding, but negatively affect mitotic progression by altering spindle polarity.

## Results

### MCTS growth in conditions of mechanical confinement

**Figure 1:**
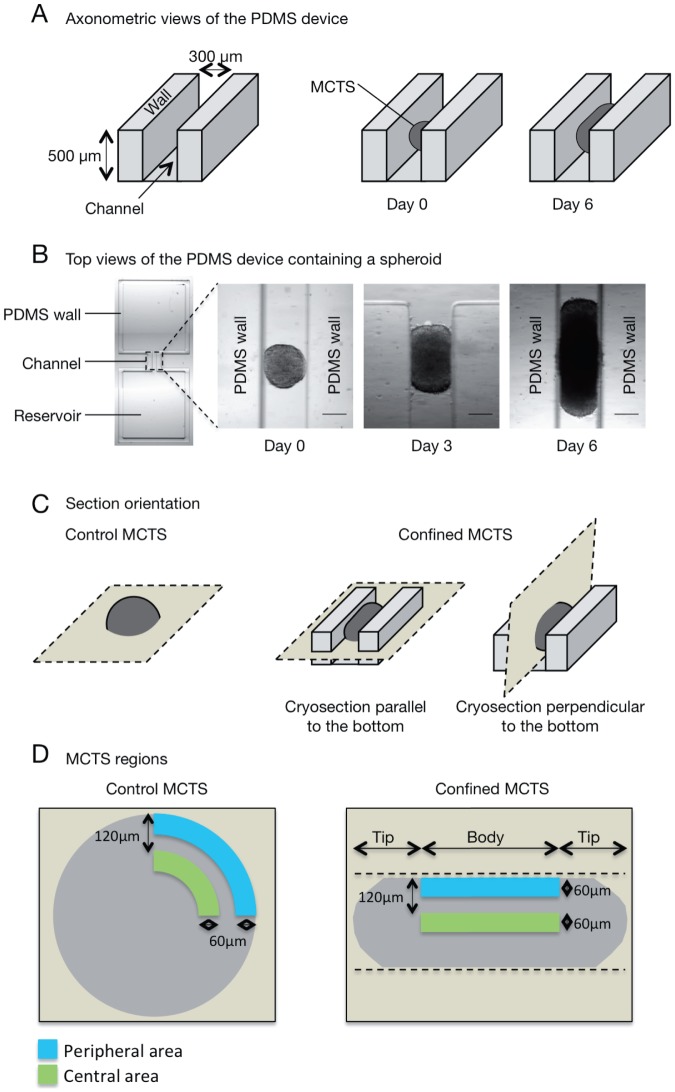
Experimental setup with PDMS devices. (A) Schematic representations (axonometric view) of the PDMS microdevice (left panel), of a spheroid that has just been placed in the PDMS microdevice (middle panel) and after 6 days of culture (right panel). (B) Transmitted-light images (top view) of a PDMS microdevice (left panel) and of a spheroid at day 0, day 3 and day 6 of growth within the microdevice (scale bar, 200 µm). (C) Schematic representation of how control MCTS (grown in non-confined conditions) and mechanically confined MCTS (grown in the PDMS microdevice) were oriented for cryosectioning. (D) Schematic representations of sections of control and confined MCTS. The dashed lines indicate the PDMS walls. The peripheral area corresponds to a 60 µm-width region along the surface of control MCTS or along the body walls of confined MCTS. The central area corresponds to a 60 µm-width region 120 µm away from the surface in control MCTS and from the PDMS walls in the body of confined MCTS.

**Figure 2:**
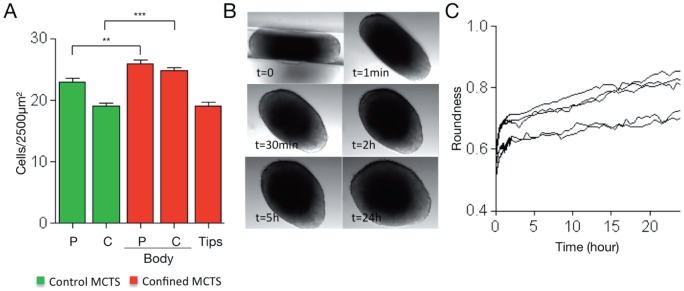
MCTS grown in PDMS microdevices are mechanically stressed. (A) Cell density in the peripheral (P) and central (C) areas of control MCTS (48 ROI analyzed from 15 MCTS obtained in 3 independent experiments) and in the body (56 ROI analyzed), and tips (39 ROI analyzed) of 6 confined MCTS from 3 independent experiments. The bars correspond to the mean ± SEM. (B) Transmitted-light images of an MCTS were acquired at the indicated times after removal from the PDMS microdevice in which it was cultured for 6 days. Images are extracted from the time-lapse experiment (Zeiss Axiovert microscope) shown in Movie S1. (C) Change in roundness values of 5 MCTS (from 5 independent experiments) over time after removal from the PDMS microdevice in which they were cultured for 6 days. The roundness values were calculated as 4x(area)/(πx(major axis)^2^) at each time point up to 24 hours after removal. A rapid swelling of the MCTS is observed just after the removal and then roundness increases more slowly.

To evaluate the impact of mechanical confinement on MCTS growth, HCT116 colorectal cancer cell spheroids of 300 µm in diameter were transferred in especially designed channel-shaped PDMS microdevices (see Fig. 1 for a description of the experimental system). In these confined culture conditions, MCTS progressively elongated as they grew within the channel of the PDMS device and acquired a rod-shaped morphology (Fig. 1B). Cell density (number of cells/µm2) was higher in the body (peripheral and central areas), but not in the tips, of confined spheroids compared to control MCTS (Fig. 2A and Fig. 1 D for a schematic description of the spheroid areas). As increased cell density has been reported in multicellular spheroids subjected to solid stress [12], we asked whether MCTS grown in confined conditions were mechanically stressed. Thus, MCTS were removed from the PDMS microdevice and their shape analyzed by time-lapse microscopy over time. Following removal from the PDMS microdevice, rod-shaped MCTS immediately relaxed and very rapidly acquired the round shape of control spheroids (Fig. 2B, C and Movie S1). This result and the increased cell density in the body region of confined spheroids strongly suggest that MCTS within the microdevice walls experience growth-associated mechanical stress.

**Figure 3:**
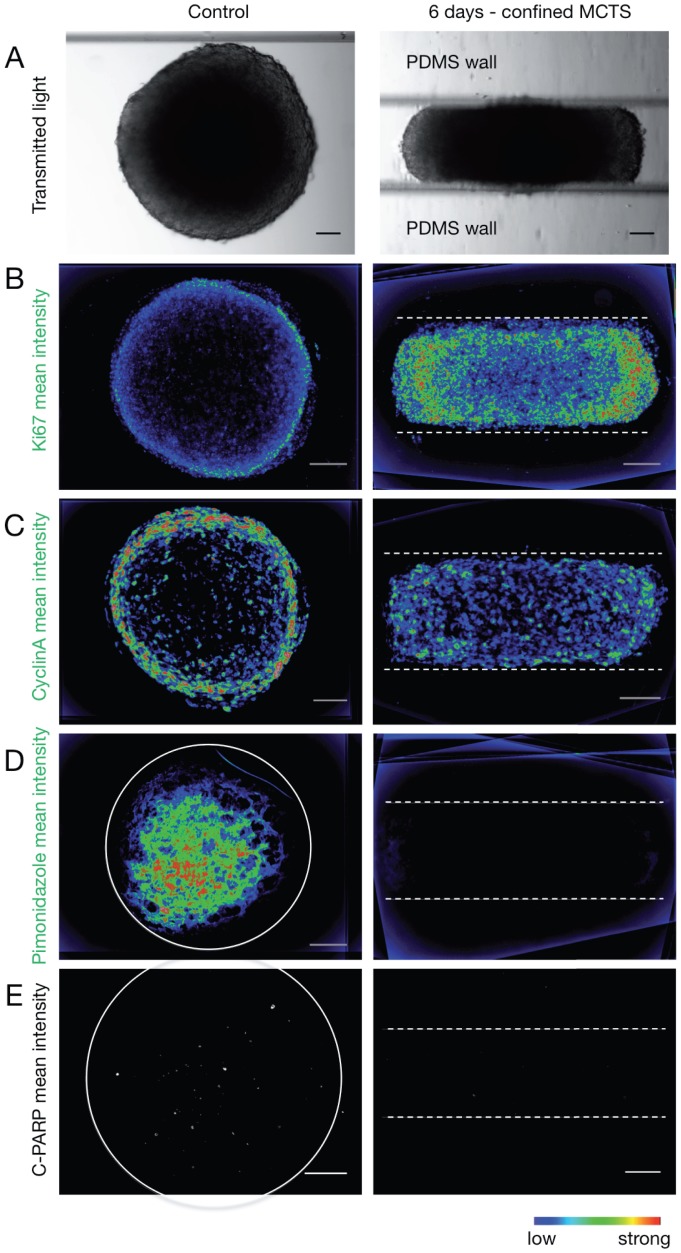
Proliferation, hypoxia and apoptosis within MCTS grown in PDMS microdevices. (A) Transmitted light images of control and confined MCTS grown in a PDMS microdevice for 6 days. (B) Detection by immunofluorescence of Ki67 staining (marker of proliferative cells). Mean fluorescence intensity of 10 cryosections from 4 different control MCTS (from 3 independent experiments) and of 6 cryosections from 4 mechanically confined MCTS (6 days in the PDMS microdevice; from 3 independent experiments). (C) Detection by immunofluorescence of Cyclin A staining. Mean fluorescence intensity of 6 cryosections from 4 different control MCTS (from 3 independent experiments) and of 6 cryosections from 4 mechanically confined MCTS (from 3 independent experiments). (D) Detection by immunofluorescence of hypoxia (pimonidazole; in green). Mean fluorescence intensity of 8 cryosections from 8 different control MCTS (from 3 independent experiments) and of 8 cryosections from 6 mechanically confined MCTS (from 4 independent experiments). The grey circle indicates the MCTS margins. (E) Cleaved PARP staining (apoptosis marker). Mean fluorescence intensity of 6 cryosections from 6 control MCTS (two independent experiments) and of 6 cryosections from 6 mechanically confined MCTS (6 days in the PDMS device; 4 independent experiments). c-PARP, cleaved PARP. The grey circle indicates the MCTS margins. (A–E) The dashed lines indicate the PDMS walls. (A–D) The color scale indicates the fluorescence intensity (scale bar, 100 µm.). Nuclei are stained by DAPI (blue).

We then analyzed the impact of growth-induced mechanical confinement on cell proliferation by immunodetection of the proliferative marker Ki67 (Fig. 3B and S1B) in MCTS cryosections that were parallel to the bottom of the channel (as depicted in Fig. 1C). In confined MCTS, Ki67-positive cells were present in the whole spheroid, but for a small area in the center. Conversely, in control MCTS grown in suspension, Ki67 staining was restricted to the outmost cell layers, as expected. Fluorescence intensity profiles (Figure S1F) confirmed these observations. Similar results were also obtained for Cyclin A, a marker of cells progressing to the S- and G2-phase of the cell cycle (Fig. 3C and S1C & F). This finding indicates that, in mechanically confined growth conditions, proliferative cells are found even in the most inner cell layers of rod-shaped MCTS. This was confirmed also by the absence of hypoxia (detected by pimonidazole staining) and of apoptotic cells (detected by cleaved PARP staining) in mechanically confined MCTS in comparison to control MCTS (Fig. 3D, E and S1D & E).

### In mechanically confined MCTS, mitotic cells are evenly distributed in the entire spheroid structure

**Figure 4:**
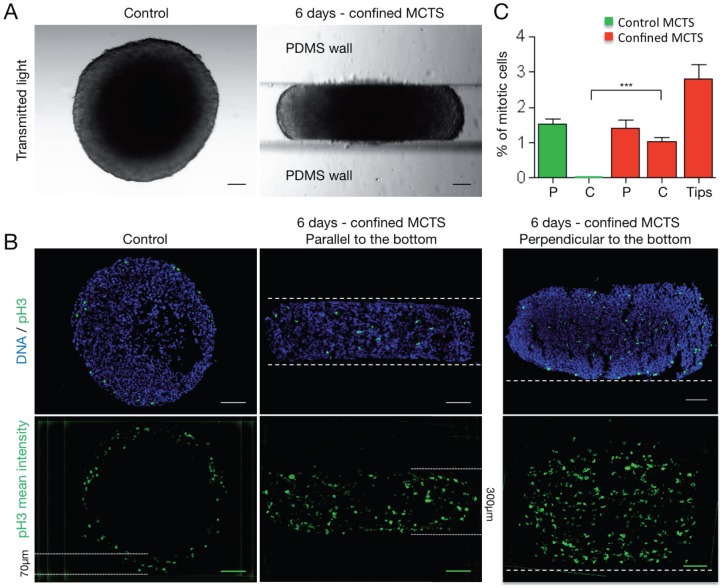
Mechanically confined growth impairs the regionalization of mitotic cells in MCTS. (A) Transmitted light images of a control MCTS and of a MCTS grown in a PDMS microdevice for 6 days. (B) Upper panels: Detection by immunofluorescence of mitotic cells (anti-phosphorylated Histone H3 antibody, pH3; in green) in cryosections of a control MCTS and a mechanically confined MCTS. The orientation of mechanically confined MCTS cryosections is parallel to the bottom of the channel (middle, see also Fig 1C) and perpendicular to the bottom of the channel (right, see also Fig 1C). Nuclei are stained with DAPI (blue). Lower panels: mean fluorescence intensity of pH3 staining in 8 cryosections from 6 control MCTS (3 independent experiments), 11 parallel cryosections from 11 mechanically confined MCTS (4 independent experiments) and 8 perpendicular cryosections from 6 mechanically confined MCTS (3 independent experiments). Dashed lines represent the walls of the PDMS channel. White lines indicate the width of the area where mitotic cells are localized (scale bar, 100 µm). (C) Percentages of mitotic cells (pH3-positive cells) in the peripheral (P) and the central (C) areas of control MCTS (n = 14 areas analyzed, from 7 MCTS from 3 experiments) and in the peripheral (P) and central (C) areas and the tips (Tips) of confined MCTS (n = 29 areas analyzed, from 12 MCTS from 6 experiments). The bars correspond to the mean ± SEM.

To characterize the effect of growth-induced mechanical stress on cell division within MCTS, the expression of phosphorylated Histone H3 (pH3; a marker of mitotic cells) was investigated after 6 days of growth in control or confined conditions (Fig. 4A). In control MCTS, pH3-positive mitotic cells were restricted to the outer cell layers (within the external 70 µm) (Fig. 4B), in accordance with the existence of a proliferative gradient [18] (Fig. 3B,C). Conversely, in mechanically confined MCTS (cryosections parallel to the bottom of the microdevice), pH3-positive mitotic cells were homogeneously distributed in the entire body of the spheroid structure (i.e., up to 150 µm away from the microdevice walls and in the spheroid body) (Fig. 4B). Similar results were obtained also using sections that were cut perpendicularly to the bottom of the PDMS microdevice through the center of the MCTS (Fig 1C and 4B). This observation was confirmed by quantifying the percentage of mitotic cells (Fig. 4C) in the peripheral (P) and central (C) areas of control and confined spheroids (see Fig. 1D for a schematic representation of these areas). These data indicate that spheroid growth in confined conditions is associated with loss of mitotic cell regionalization.

### Mechanical stress induces mitotic arrest in MCTS

**Figure 5:**
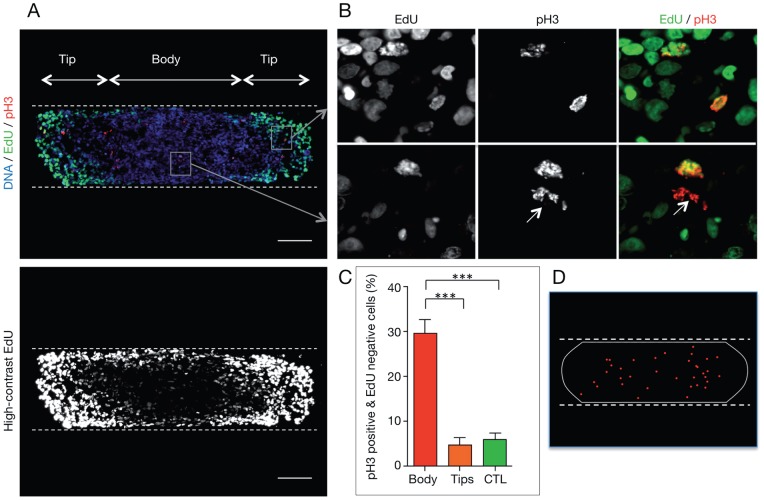
Growth-associated external mechanical stress leads to accumulation of cells arrested in mitosis. (A) Upper panel: Immunodetection of EdU incorporation (green) and mitotic cells (pH3-positive, red) in a cryosection from MCTS grown in PDMS microdevices for 6 days. Nuclei are stained using DAPI (blue). Lower panel: High-contrast image of the immunodetection of EdU incorporation (white) (scale bar, 100 µm). (B) Analysis of EdU incorporation in mitotic cells. Images correspond to magnifications of the regions indicated by the white squares in A from the tip (top panels) and the body (bottom panels) of a mechanically confined MCTS. The white arrow indicates a pH3- positive cell that is not EdU-positive. This cell is next to a pH3-positive/EdU-positive cell. (C) Percentage of pH3-positive/EdU-negative cells in the body (589 mitotic cells from 48 cryosections) and in the tips (331 mitotic cells from 95 cryosections) of mechanically confined MCTS (20 MCTS from 4 independent experiments) and in control (CTL) MCTS (358 mitotic cells from 34 cryosections from 15 MCTS from 4 independent experiment). Bars correspond to the mean ± SEM. (D) Map showing the localization of pH3-positive/EdU-negative cells in 8 cryosections from 8 mechanically confined MCTS from 4 independent experiments. The white line represents the outline of the MCTS and the red dots the localization of the pH3-positive/EdU-negative cells. The dashed lines indicate the microdevice PDMS walls.

Accumulation of mitotic cells in mechanically confined MCTS could result from enhanced cell proliferation or from mitotic arrest. To explore these hypotheses, control and mechanically confined MCTS were incubated with EdU for 24 hours prior to fixation (total culture time  = 6 days). Preliminary experiments determined that this incubation time was required to ensure that EdU was incorporated by the majority of proliferating cells during DNA replication (data not shown). Analysis of EdU incorporation in cryosections parallel to the bottom of the microdevice and passing through the center of mechanically confined MCTS (Fig. 5A) indicated that EdU-positive cell distribution is comparable to that of Ki67-positive cells (see high-contrast image in the bottom panel of Fig. 5A), showing that EdU was well incorporated and confirming that proliferating cells are present everywhere in mechanically stressed MCTS.

In these experimental conditions, mitotic cells (as indicated by pH3 staining) that underwent S-phase during the last 24 hours should have incorporated EdU. However, while in the tips of mechanically confined MCTS most pH3-positive cells were also EdU-positive, in the body of confined MCTS a number of pH3-positive cells were EdU-negative (Fig. 5A, B). This was not due to incomplete EdU penetration in the MCTS body, because EdU/pH3 double positive cells were detected in proximity of pH3-positive cells that did not incorporate EdU (Fig. 5B, arrow). Quantification of the percentage of pH3-positive/EdU-negative cells in control and confined MCTS showed that nearly 30% of mitotic cells were EdU-negative in the body of rod-shaped MCTS, while only about 5% of pH3-positive cells were EdU-negative in control spheroids and at the tips of mechanically stressed MCTS (Fig. 5C). This highly significant difference (p<0.0001) indicates that EdU-negative mitotic cells (pH3 positive) had been in G2- or M-phase for at least 24 hours prior to fixation. Moreover, quantification of EdU-negative/pH3-positive cells in MCTS after 4 days of growth in confined conditions showed that only 11.4% of pH3-positive cells were EdU-negative in the spheroid body (data not shown). Analysis of the location of mitotically arrested cells within mechanically stressed MCTS showed that they were mostly found within the body of the spheroid, both in the peripheral and central regions (Fig. 5D). These findings indicate that pH3-positive/EdU-negative cells are progressively accumulated during growth in the confined environment of the PDMS microdevice and strongly suggest that growth-induced mechanical stress impairs mitotic progression in the body region of the MCTS.

### External mechanical stress impairs bipolar spindle assembly

**Figure 6:**
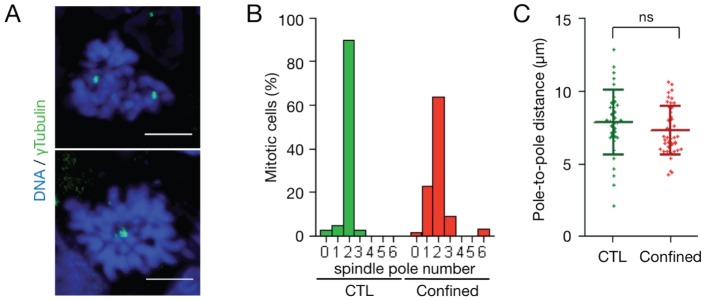
Mechanically confined MCTS show bipolar spindle defects. (A) Maximal projection of two mitotic cells from two z-stacks of images of a cryosection from a mechanically confined MCTS (6 days in the PDMS microdevice) incubated with an anti-γTubulin antibody (green). Nuclei were stained with DAPI (blue) (scale bar, 5 µm). (B) Distribution (percentage) of mitotic cells as a function of the number of spindle poles in control MCTS (CTL, 37 mitotic cells analyzed from 10 MCTS from 3 independent experiment) and in the body of mechanically confined MCTS (Confined, 66 mitotic cells analyzed from 8 MCTS from 4 independent experiment). (C) Distribution of the pole-to-pole distance (in µm) in bipolar mitotic cells from the control (CTL) and mechanically confined (Confined) MCTS analyzed in (B). The lines correspond to the mean ± SD.

Establishment of a bipolar spindle is a prerequisite to achieve even mitotic segregation of chromosomes in daughter cells. When mitotic spindles are not correctly assembled, the mitotic checkpoint is activated and cells cannot undergo the metaphase-anaphase transition, leading to mitotic arrest [19]. To determine whether mitotic spindle bipolarity was affected in confined spheroids, the number of spindle poles in entire mitotic cells within MCTS was determined. To this aim, expression of γTubulin, a spindle pole component, was assessed in MCTS cryosections thick enough to identify entire cells (Fig. 6A). Confocal microscopy allowed the 3D visualization of spindle poles and chromosomes (Fig. 6A). In control MCTS, 89.7% of mitotic cells had two spindle poles. In contrast, in the body of mechanically confined spheroids only 63.6% of mitotic cells had two spindle poles, 22.7% only one and 9.1% three (Fig. 6B). The pole-to-pole distance was similar in cells from control and confined spheroids when two poles were detected (Fig. 6C). This result indicates that in spheroids grown in a confined environment, mitotic spindle bipolarity establishment is locally impaired.

### Growth in confined conditions does not affect mitotic cell rounding

**Figure 7:**
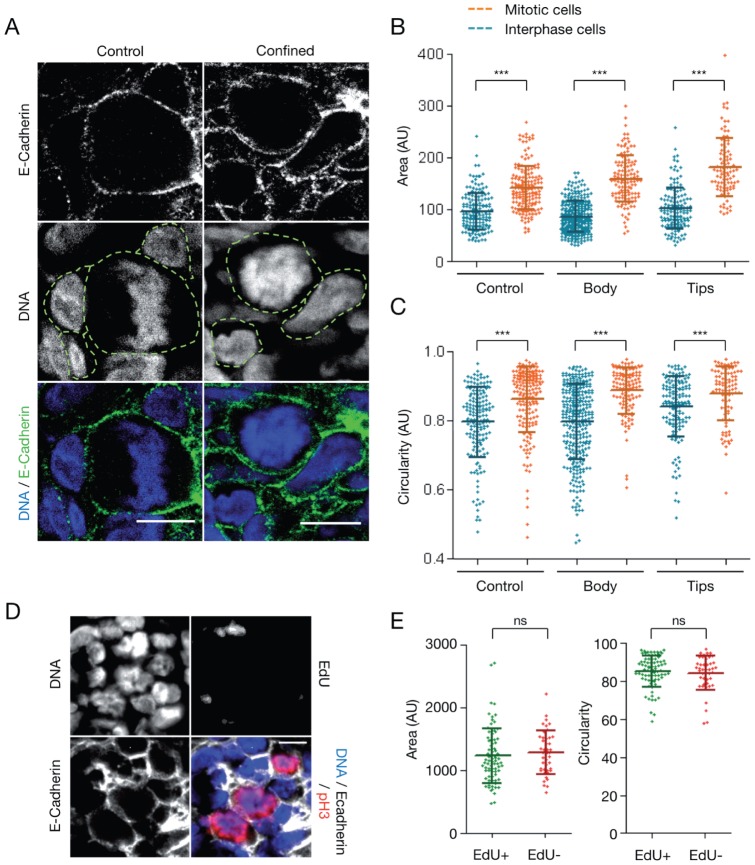
Mechanical confinement does not impair mitotic cell rounding within MCTS. (A) Cryosections of a control MCTS and a mechanically confined MTCS (6 days in the PDMS device), stained for DNA (blue) and E-Cadherin (green) (scale bar, 10 µm). The cell outlines are drawn manually (green dashed line) to extract the area and the circularity of cells. (B) Area values of interphase cells (blue) and mitotic cells (orange) in control MCTS and in the body and tips of mechanically confined MCTS (6 days in the PDMS microdevice). Lines correspond to the mean ± SD. (C) Circularity values of interphase cells (blue) and mitotic cells (orange) in control MTCS and in the body and tips of mechanically confined MCTS (6 days in the PDMS microdevice). The error bars represent the mean ± SD. For control MCTS, 167 interphase cells and 175 mitotic cells were analyzed from 8 MCTS from 2 experiments. For confined MCTS, 307 interphase cells and 125 mitotic cells were analyzed in the body, and 146 interphase cells and 91 mitotic cells were analyzed in the tip, both from 10 MCTS from 4 experiments. (D) Cryosections of a mechanically confined MCTS stained for DNA (blue), E-Cadherin (grey), EdU (green) and pH3 (red) (scale bar, 10 µm). (E) Area (left panel) and circularity (right panel) values of pH3-positive mitotic cells in the body of mechanically confined MCTS relative to EdU incorporation (EdU+/pH3+: 79 cells analyzed, EdU−/pH3+: 43 cells analyzed, from 3 MCTS from 2 independent experiments). The lines correspond to the mean ± SD.

As spindle defects and mitotic progression delay could be caused by failure of cells to round up [20], the morphology of mitotic cells was analyzed by incubating thick cryosections of control and mechanically confined MCTS with antibodies against pH3 and E-cadherin to identify the contours of entire mitotic cells (Fig. 7A). The area and circularity of interphase and mitotic cells were measured (Fig. 7B and C). Overall, mitotic cells had circularity values higher than interphase cells in both control and confined MCTS. Moreover, the circularity values of mitotic cells were similar in both conditions, demonstrating that, in multicellular tumor models, cells round up when they undergo mitosis. This result also indicates that mechanical confinement does not impair cell rounding at mitosis. Then, to test if rounding could be specifically impaired in mitotically arrested cells, cells were labeled with EdU to detect pH3-positive/EdU-negative cells as before (Fig. 7D). No significant difference in the area and circularity values of pH3-positive/EdU-negative and pH3-positive/EdU-positive cells was observed in confined MCTS (Fig. 7E). These data clearly demonstrate that growth-induced mechanical stress does not prevent mitotic cell rounding within the MCTS body and indicate that mitosis arrest and bipolar spindle assembly impairment in mechanically stressed MCTS are not due to a defect in cell rounding at mitosis.

## Discussion

In addition to growth-induced solid or stored stress, which are generated by cell and matrix tumor components, reciprocal forces from the surrounding environment opposed to tumor growth also can contribute to the stress experienced by a tumor cell population. This type of stress can be designed as growth-associated (induced makes one think that is the growth causing the stress, whereas in reality is the external “obstacle” that limits growth) external mechanical stress.

In this study, we investigated the impact of a confined mechanical environment on cell proliferation in micro-tumors and our findings suggest that growth-associated external mechanical stress impairs mitosis progression.

As a paradigm we used MCTS, a model that accurately reproduces the 3D organization of a micro-tumor in vitro [16]. Studies devoted to the exploration of tissue mechanical properties have been performed on spheroids that were mechanically stressed by using parallel-plate tensiometry [21], [22] or micropipette aspiration [23]. Various parameters such as surface tension or viscosity could be recorded, but very few studies addressed the impact of solid stress on cell proliferation within spheroids. Helmlinger and coworkers showed that isotropic solid stress produced by embedding MCTS within an agarose matrix led to spheroid growth inhibition with decreased apoptosis and slight reduction of cell proliferation [12]. In similar experiments, Cheng and collaborators reported that inhomogeneity in the mechanical properties of the confining environment could lead to morphological changes during tumor growth by inducing apoptosis in spheroid regions that are subjected to high compressive stress and by promoting proliferation in low-stress regions [11]. These studies indicated that solid stress influences tumor growth at both macroscopic and cellular levels. In the work reported here we attempted to explore further the impact of a confined mechanical environment on spheroids growth. To this aim, we analyzed by immunohistochemistry the MCTS cell response when grown in a dedicated PDMS microdevice we developed to generate growth-associated mechanical stress. After 6 days of culture within the PDMS channel, spheroids adopted an elongated rod shape with their longer axis parallel to the channel axis, in agreement with the previous reports [11], [12]. This growth directionality was accompanied by modification of the hypoxia and proliferation gradients. Cell morphology data show that growth-associated mechanical stress under confinement induces modifications of the cell shape and organization within the body and the tip regions of spheroids. It is known, from various biological models, that cell geometry and organization is modified in response to mechanical forces occurring during growth and morphogenesis [24]. These cell morphology parameters are closely related to cell-cell contacts and cell-cell signaling, which are important for the mechanical regulation of cell physiology. In the spheroid model, modification of cell density could affect cell-cell contacts and this might contribute to the specific cell features observed in confined spheroids. More advanced cellular and molecular techniques are required to investigate this hypothesis.

In addition and strikingly, growth-associated mechanical stress resulted in mitotic defects. Cell division is a crucial step and its accuracy relies on spatially and temporally tightly controlled mechanisms. Positioning of the mitotic cleavage plane depends on the interaction of the cell with the extracellular environment [25] and with neighboring cells [26]–[28]. Moreover, several studies showed that cell rounding, a common feature of cell division that relies on hydrostatic pressure and cell cortex forces [29], is a key parameter for correct mitosis progression [30]–[32]. As cell shape and interaction with the microenvironment rely on and can be modeled by forces, all these data emphasize the importance of mechanical cues on cell division. Mechanical confinement has been shown to impair mitotic cell rounding and to induce aberrant cell division [33], [34]. However, all these results were obtained using 2D cell models or simple epithelia, and little is known about the impact of such mechanical stress on cell division control within tumors. The present study demonstrates that mitotic cell rounding is not impaired within tumor spheroids cultured in growth-associated stress conditions and that therefore it might not be involved in the observed mitotic progression delay and mitotic spindle defect. This result is not in agreement with a recent report that shows a strong interplay between cell rounding at mitosis and mitotic progression [33]. However, in this study isolated cells were used, whereas we analyzed this interplay in a multicellular model and in experimental conditions that mimic the behavior of a growing micro-tumor in an anisotropic mechanical environment resulting from tissue stiffness differences. On the other hand, in such 3D model it is more difficult to identify the involved molecular players. Understanding the discrepancies between our results and previous literature data will require functional studies based on loss-of-function experiments using pharmacological or siRNA approaches that are currently under development.

## Materials and Methods

### Cell culture and spheroid generation

HCT116 colorectal cancer cells (ATCC) were cultured in DMEM (Invitrogen) containing 10% FCS with 2 mM/l glutamine and penicillin/streptomycin in a humidified atmosphere of 5% CO2 at 37°C. Spheroids were prepared as previously described [18], [35]. Briefly, 500 cells/well were distributed in poly-HEMA-coated 96-round bottom well plates. Plates were centrifuged (300 g for 6 min) and then placed in a humidified atmosphere of 5% CO2 at 37°C.

### PDMS device

Polydimethylsiloxane (PDMS) is gas-permeable polymer, non-toxic and considered as inert. We designed a channel-shape device (Fig. 1) with reservoirs on both side to ensure homogeneous feeding of the spheroids. PDMS was first degassed in a vacuum chamber then poured on a silicium wafer (LAAS technical facility). After a second degassing, PDMS was heated at 80°C for 6 hours. PDMS was then peeled off from the wafer to make the molded devices in which spheroids were transferred when they measured about 300 µm in diameter.

### Immunofluorescence on cryosections

Spheroids in free suspension or in PDMS microdevices were fixed in formalin (Sigma) for 2–3 hrs, then washed with PBS and stored at 4°C. After fixation, spheroids alone or within the PDMS microdevice were incubated in 15% and then 30% sucrose in PBS at 4°C for 24 h, embedded in Tissue-Tek (Sakura Finetek) and then 7 µm- or 15 µm-thick cryosections were cut. After blocking in PSB/1% BSA/0.5% Triton, sections were incubated with antibodies against Ki67 (rabbit polyclonal, Santa Cruz, 1/200 at 4°C, overnight), cleaved PARP (rabbit monoclonal, Epitomics, 1/1000 at 37°C, for 1 h), phosphorylated HistoneH3 (rabbit polyclonal, Millipore, 1/2000 at 37°C, for 1 h), E-Cadherin (mouse polyclonal, Abcam, 1/200 at 4°C, for 72 h) or γTubulin (mouse polyclonal, Sigma, 1/5000 at 4°C, for 72 h). After washes in PBS/0.1% Triton v/v, the secondary antibody was added for 1 h (anti-mouse or anti-rabbit conjugated with Alexa 488, Alexa 594 or Alexa647, Molecular Probes, 1/800, at room temperature). DNA was stained using DAPI. An initial antigen retrieval step (boiling in a solution containing 2 mM citric acid and 8 mM sodium citrate for 3×7 mn) was included for the anti-Ki67 and -E-Cadherin antibodies.

For hypoxia detection, spheroids were incubated at 37°C with 100 µM pimonidazole for 2 hours prior to fixation. The hypoxic regions were revealed on cryosections by incubation with the FITC-conjugated MAb1 for 2 h (Hypoxyprobe™-1 Plus Kit, HPI; 1/300 at 37°C).

### EdU labeling of spheroids

EdU labeling was performed by using the Click-iT® EdU Alexa Fluor® Imaging Kit (Molecular Probes). EdU (5-ethynyl-2′-deoxyuridine) is a thymidine analog that is incorporated into newly synthesized DNA. EdU was added to the culture medium to a final concentration of 10 µM. After 24 h incubation, spheroids were rinsed in PBS and fixed. EdU detection, based on a specific click reaction between EdU and the Alexa FLuor® 488 or 594 dye, was performed following the manufacturer's instructions.

### Images acquisition and analysis

Transmitted light images of spheroids were acquired using a MacroFluo Z16 APO microscope (Leica) fitted with a CoolSNAP ES2 CCD camera (Roper). Time-lapse data of spheroids were acquired using a widefield microscope (Zeiss) with a 10X objective (NA 0.3). Roundness analyses were performed using the Cellomics Technologies software (Compartimental Bioapplication-Thermo Scientific). Briefly, the outline of the spheroid was automatically detected and its roundness was calculated by the software. Fluorescence images of 7 µm-spheroid sections were acquired using a DM5000 (Leica) epifluorescence microscope, fitted with a Roper COOLsnap ES CCD camera. Images were processed using the Metavue and ImageJ software packages. The intensity averaging corresponds to the calculation of the average intensity of each pixel in the entire stack. It results in the production of an image showing the average spatial distribution of the antibody of interest. Cell counting was performed manually. Fluorescence images of 15 µm-spheroid sections were acquired using a LSM510 NLO multiphoton confocal microscope (Carl Zeiss, Jena, Germany) and a 63X objective. Imaging of immunofluorescence experiments involved the sequential use of 488- and 594-nm lasers and an IR pulsed laser at 750 nm for DAPI. Images were processed using the Zen 2009 software (Zeiss). Cell shape and cell density analyses were performed using ImageJ plugins. The cell outlines were drawn manually and the ROI geometrical parameters were given by the software. The density analyses were performed by counting the number of cell in ROI of 2500 µm^2^. Cell polarity analyses were done using the Imaris 7.0.0 software (Bitplane).

### Statistical analyses

Distribution, histograms and the Wilcoxon Mann-Whitney test were performed using the Microsoft Excel and Prism software packages.

## Supporting Information

Figure S1 **Proliferation, hypoxia and apoptosis within MCTS grown in PDMS microdevices.** (A) Transmitted light images of a control MCTS and a MCTS grown in a PDMS microdevice for 6 days. (B) Detection by immunofluorescence of proliferative cells (Ki67; in green) in cryosections from a control MCTS and a mechanically confined MCTS (6 days in the PDMS device); nuclei are stained by DAPI (blue) (representative fluorescence image). (C) Detection by immunofluorescence of cells in the G1 phase of the cell cycle (Cyclin A; in red) in cryosections from a control MCTS and a mechanically confined MCTS (6 days in the PDMS device). Nuclei are stained by DAPI (blue) (representative fluorescence image). (D) Detection by immunofluorescence of hypoxia (pimonidazole; in green) in cryosections from a control MCTS and a mechanically confined MCTS (6 days in the PDMS device). Nuclei are stained by DAPI (blue) (representative fluorescence image). (E) Detection by immunofluorescence of apoptotic cells (c-PARP; in red) in cryosections from a control MCTS and a mechanically confined MCTS (6 days in the PDMS device). Nuclei are stained by DAPI (blue) (representative fluorescence image). Scale bar, 100 µm. The dashed lines indicate the PDMS walls. (F) Plot profiles of the mean Ki67 (top) and Cyclin A (bottom) fluorescence intensity from Figure 3B and C, respectively. For each antibody, the fluorescence intensity corresponded to the average intensity in a 50 µm-wide ROI spanning along the diameter of control MCTS (green) or along the major axis (red) of mechanically confined MCTS, as shown in the schematic representation of MCTS (upper panel).
(EPS)

Movie S1 **MCTS removal from the PDMS microdevice.** Time-lapse analysis (image acquisition in transmitted-light) of a spheroid removed from the PDMS microdevice and grown in suspension (Zeiss Axiovert microscope, 10X objective). The movie starts one minute after the removal from the microdevice and covers a period of 5.5 hours.
(AVI)
